# A population-weighted, condition-adjusted estimate of palivizumab efficacy in preventing RSV-related hospitalizations among US high-risk children

**DOI:** 10.4161/hv.32082

**Published:** 2014-11-21

**Authors:** Christopher S Ambrose, Xin Chen, Veena R Kumar

**Affiliations:** AstraZeneca; Gaithersburg, MD USA

**Keywords:** palivizumab, efficacy, RSV-related hospitalization, population-weighted efficacy, condition-adjusted efficacy, respiratory syncytial virus, severe RSV disease

## Abstract

Preterm infants ≤ 35 weeks' gestational age (GA), and children ≤ 24 months of age with bronchopulmonary dysplasia (BPD) or hemodynamically significant congenital heart disease (hsCHD) are at high risk for developing severe respiratory syncytial virus (RSV) disease. In 3 previous randomized, placebo-controlled trials, palivizumab efficacy varied significantly based on these underlying conditions, and trial enrollment was not proportional to condition prevalence. This analysis provides the first estimate of the population-weighted efficacy of palivizumab in high-risk children, adjusting for condition prevalence. Palivizumab efficacy by high-risk condition was obtained from the clinical trials. The annual number of US children with each condition was obtained from the 2010 Centers for Disease Control and Prevention (CDC) natality statistics and the medical literature. Data from specialty pharmacies in the US palivizumab distribution network were used to estimate the population for each condition receiving at least 1 dose in the outpatient setting in 2012–2013. The weighted efficacy estimate was derived by summing the products of the condition-specific relative risk reductions and the relative frequency of each condition among those receiving palivizumab. The US population-weighted efficacy estimate for those receiving palivizumab was 68%. Due to the low prevalence of BPD and hsCHD and the higher efficacy observed in preterm infants without BPD or CHD, the population-weighted estimate of palivizumab efficacy is higher than the overall 45–55% efficacy observed in initial clinical trials. Consistent with 2012 American Academy of Pediatrics RSV prophylaxis recommendations, a low proportion of preterm infants 32–35 weeks' gestational age receive palivizumab.

## Abbreviations

BPD: bronchopulmonary dysplasia
CDC: Centers for Disease Control and Prevention
CHD: congenital heart disease
FDA: US Food and Drug Administration
GA: gestational age
hsCHD: hemodynamically significant congenital heart disease
RSV: respiratory syncytial virus
RSVH: respiratory syncytial virus-related hospitalization

## Introduction

Respiratory syncytial virus (RSV), an important pathogen of infants and young children, is the most common cause of bronchiolitis and pneumonia worldwide.^1^ Severe RSV disease causes significant morbidity and is the leading cause of hospitalization among infants in the United States (US).^2-4^ Preterm infants ≤ 35 wk gestational age (GA), and children ≤24 mo of age with bronchopulmonary dysplasia (BPD)^5^ or hemodynamically significant congenital heart disease (hsCHD) are at high risk for developing severe RSV disease.^6^

Palivizumab is a humanized monoclonal antibody indicated for the prevention of serious lower respiratory tract disease in these high-risk populations.^7^ Common side effects of palivizumab include fever and rash, and children should not receive palivizumab if they have ever had a severe allergic reaction to palivizumab.^7^ In 3 previous randomized, placebo-controlled trials,^5,6,8^ palivizumab efficacy varied significantly based on the presence of the aforementioned underlying high-risk conditions. Additionally, trial enrollment was not proportional to condition prevalence; for example, a trial of preterm infants with or without BPD overrepresented those with BPD. Palivizumab was shown to reduce RSV-related hospitalizations (RSVH) in children ≤ 24 mo of age at RSV season start with BPD or hsCHD by 39% (95% CI: 20, 58) and 45% (95% CI: 23, 67), respectively.^5,6^ Among preterm infants ≤ 35 wk GA without BPD, palivizumab reduced RSVH by 72% (95% CI: 17.4, 90.8) among <32-wk GA infants and by 82% (95% CI: 45.4, 94.2) among 32- to 35-wk GA infants.^5^ A recent randomized clinical trial also demonstrated that the efficacy of palivizumab in reducing RSVH among 32- to 35-wk GA infants without BPD was 82% (95% CI: 18, 157).^8^ Lastly, utilization of palivizumab also varies significantly by condition based on recommendations for use.

This analysis provides the first estimate of the population-weighted efficacy of palivizumab in US children at high risk for severe RSV disease, adjusting for condition prevalence.

## Materials and Methods

Palivizumab efficacy by specific high-risk condition (ie, preterm infants ≤35 wk GA and children ≤24 mo of age with BPD or hsCHD) was obtained from the results of the following 3 clinical trials.^5,6,8^ The IMpact-RSV study was a multicenter, randomized, double-blind, placebo-controlled trial that enrolled preterm infants born at ≤35 wk GA who were ≤6 mo of age and children ≤24 mo of age with BPD.^5^ The multicenter, randomized, double-blind, placebo-controlled study conducted by Feltes et al.^6^ enrolled children ≤ 24 mo of age with hsCHD. Blanken et al.^8^ conducted a double-blind, placebo-controlled study that included preterm infants 32–35 wk GA ≤ 6 mo of age who were otherwise healthy. Study procedures were conducted in accordance with the principles of the Declaration of Helsinki, the US Code of Federal Regulations for protection of human subjects, and Institutional Review Boards.^5,6,8^

The annual number of US infants with prematurity by GA subgroup was obtained from the 2010 Centers for Disease Control and Prevention (CDC) natality statistics.^9^ Estimates of the number of children with BPD or moderate/severe CHD were obtained from the medical literature.^10-12^ The estimated prevalence of BPD by GA subgroup was 25.4% of infants <32 wk GA^12^; 1.5% of infants 32–33 wk GA^10^; and 0.34% of infants 34–35 wk GA.^10^ The estimated number of children with moderate or severe CHD was 0.6%.^11^ Estimates of the number of preterm infants without BPD or moderate/severe CHD were obtained by subtracting the estimates for moderate/severe CHD and the GA-specific estimates of BPD from the number of births reported in the CDC natality statistics. For estimates of the populations eligible to receive palivizumab, no data were available regarding the percentage of children with BPD requiring oxygen, steroids, diuretics, or bronchodilators in the 6 mo before the start of RSV season; the percentage of children with moderate or severe CHD who have hsCHD; or the percentage of infants 32–35 wk GA with American Academy of Pediatrics (AAP) 2012 risk factors (ie, attends child care or has ≥1 older child <5 y of age living in the same household; excluding multiple-birth siblings < 1 y of age).^13^

Data from specialty pharmacies in the US palivizumab distribution network were used to estimate the population for each condition receiving ≥ 1 dose of palivizumab in the outpatient setting in 2012–2013. The weighted efficacy estimate was derived by summing the products of the condition-specific relative risk reductions from the clinical trials (**Table 1**) and the relative frequency of each condition among those receiving palivizumab.

**Table 1.** Population-Weighted Efficacy of Palivizumab in Children at High Risk for Severe RSV Disease

Underlying Condition	Estimated Population Size	Estimated Annual Population Receiving Palivizumab (% of Population)	Condition-Specific Relative Risk Reduction in RSV-Related Hospitalization, % (*P* Value)
Children ≤24 mo with BPD	43,000*	10,900 (25)	39 (0.038)
Children ≤24 mo with moderate/severe CHD	19,300^†^	6600 (34)	45 (0.003)
Preterm infants <32 wk GA and age ≤6 mo without BPD or CHD	,44,800^‡^	36,500 (81)	72 (0.027)
Preterm infants 32–35 wk GA and age ≤6 mo without BPD or CHD	159,000^‡^	23,600 (15)	82^§^ (0.001)

AAP, American Academy of Pediatrics; BPD, bronchopulmonary dysplasia; CHD, congenital heart disease; wk GA, weeks' gestational age; RSV, respiratory syncytial virus; *Data source^10,12^; ^†^Data source^11^; ^‡^Data source^9^; ^§^Among preterm infants 32–35 wk GA with 2012 AAP risk factors (attends child care or has ≥1 older child <5 y of age living in the same household; excluding multiple-birth siblings <1 y of age), efficacy was consistent at 93% (RSV-related hospitalization: 12.5% for placebo vs 0.9% for palivizumab; *P* = 0.004).

## Results

**Figure 1.**
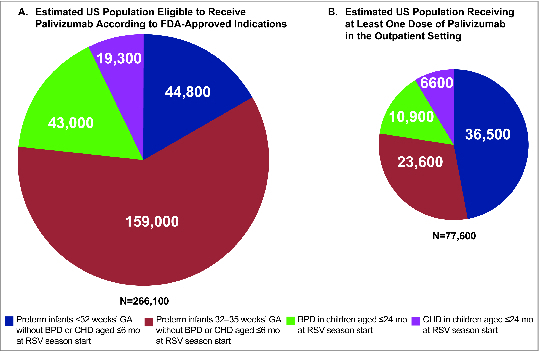
Estimated US population (**A**) eligible to receive palivizumab according to FDA-approved indication and (**B**) receiving at least one dose of palivizumab in the outpatient setting. BPD, bronchopulmonary dysplasia; CHD, congenital heart disease; FDA, US Food and Drug Administration; GA, gestational age.

The estimated total US population eligible to receive palivizumab and the estimated population receiving at least one dose of palivizumab in the outpatient setting by underlying condition is shown in **Table 1** and **Figure 1**. Overall, among the total annual US population of 250,500 high-risk children potentially eligible to receive palivizumab based on the US Food and Drug Administration (FDA)-approved indication, an estimated 31% receive at least one dose of palivizumab in the outpatient setting. For each respective condition, the proportion of children receiving palivizumab is substantially lower than the estimated population potentially eligible to receive it (**Table 1** and **Figure 1**). Depending on the condition, an estimated 15%‒67% of the eligible population receives palivizumab. Consistent with 2012 AAP recommendations for RSV prophylaxis, the lowest estimated proportion of palivizumab receipt (15%) occurs among infants 32–35 wk GA; this is 4-fold lower compared with infants <32 wk GA who have an estimated receipt proportion of 67%.

Compared with placebo, the relative risk reduction in RSVH with palivizumab was greater in preterm infants without BPD or CHD than in children with BPD or moderate/severe CHD (**Table 1**). The population-weighted efficacy estimates were 71% for those potentially eligible to receive palivizumab and 68% for those receiving palivizumab.

## Discussion

Because of the low prevalence of BPD and hsCHD and the higher efficacy observed in preterm infants without BPD or CHD, the population-weighted estimate of palivizumab efficacy is approximately 70%, which is higher than the overall 45–55% efficacy observed in the initial clinical trials. Previous studies have demonstrated the efficacy of palivizumab in helping to prevent hospitalizations due to severe RSV disease.^5,6,8^ This is the first estimate of the population-weighted efficacy of palivizumab and may provide a more accurate estimate of the cumulative efficacy among the US populations receiving palivizumab.

Based on the 2012 AAP guidelines, RSV prophylaxis is recommended throughout the RSV season for all infants born at <32 wk GA.^9^ In preterm infants 32–34 wk GA, palivizumab is recommended only through 90 d of age and only if the infant has at least 1 of the following 2 risk factors: attends child care or has ≥1 older sibling <5 y of age or other children <5 y of age who live permanently in the same household.^9^ Consistent with these recommendations, the present analysis demonstrates a substantially lower proportion of preterm infants 32–35 wk GA receive palivizumab compared with those who are < 32 wk GA.

A strength of this analysis is that it included data from 3 randomized, double-blind, placebo-controlled trials. Limitations include assumptions inherent in modeled projections and the fact that no distribution data were available for hospital utilization of palivizumab. However, hospital utilization represents a small fraction of palivizumab use as eligible infants would only receive their initial dose before discharge from the birth hospitalization during the RSV season.^13^ Additionally, as noted above, in estimating the populations potentially eligible to receive palivizumab, no data were available regarding the percentage of children with BPD requiring oxygen, steroids, diuretics, or bronchodilators in the 6 mo before the start of RSV season; the percentage of children with moderate or severe CHD who have hsCHD; or the percentage of infants 32–35 wk GA with 2012 AAP risk factors.

In conclusion, this analysis estimates the population-weighted efficacy of palivizumab in reducing RSVH at approximately 70%, which is higher than the overall 45–55% efficacy observed in initial clinical trials. The proportion of children receiving palivizumab on an annual basis is substantially lower relative to the total population that is eligible to receive it.
